# Progress towards non-small-cell lung cancer models that represent clinical evolutionary trajectories

**DOI:** 10.1098/rsob.200247

**Published:** 2021-01-13

**Authors:** Robert E. Hynds, Kristopher K. Frese, David R. Pearce, Eva Grönroos, Caroline Dive, Charles Swanton

**Affiliations:** 1 Cancer Research UK Lung Cancer Centre of Excellence, UCL Cancer Institute, University College London, London, UK; 2 Cancer Evolution and Genome Instability Laboratory, The Francis Crick Institute, London, UK; 3 Cancer Research UK Lung Cancer Centre of Excellence, University of Manchester, Manchester, UK; 4 Cancer Research UK Manchester Institute Cancer Biomarker Centre, University of Manchester, Alderley Park, Macclesfield, UK

**Keywords:** cell lines, organoids, patient-derived xenografts, genetically engineered mouse models, cancer evolution, model systems

## Abstract

Non-small-cell lung cancer (NSCLC) is the leading cause of cancer-related deaths worldwide. Although advances are being made towards earlier detection and the development of impactful targeted therapies and immunotherapies, the 5-year survival of patients with advanced disease is still below 20%. Effective cancer research relies on pre-clinical model systems that accurately reflect the evolutionary course of disease progression and mimic patient responses to therapy. Here, we review pre-clinical models, including genetically engineered mouse models and patient-derived materials, such as cell lines, primary cell cultures, explant cultures and xenografts, that are currently being used to interrogate NSCLC evolution from pre-invasive disease through locally invasive cancer to the metastatic colonization of distant organ sites.

## 1. Background

Lung cancer is the most commonly diagnosed cancer type globally for men and women, and constitutes almost one in five cancer deaths worldwide [1]. Lung cancers are classified as either small-cell lung cancer (SCLC; approx. 15%) or non-small-cell lung cancer (NSCLC; approx. 85%). NSCLC is, in turn, divisible into two main histological subtypes: lung adenocarcinoma (LUAD) and lung squamous cell carcinoma (LUSC), plus several less frequently observed subtypes, such as large cell carcinoma, adenosquamous carcinoma and carcinoid tumours. LUAD typically arises in the distal lung, whereas LUSC arises centrally, probably reflecting different cells-of-origin for these two lung cancer types [2]. It is widely thought that LUAD develops from alveolar type II (AT2) epithelial cells or cells within bronchioalveolar duct junctions, whereas LUSC develops from basal epithelial cells in airways, although data from animal models [3] and an increasing appreciation of the plasticity of lung epithelial cells [4] make this uncertain.

The two major NSCLC subtypes can be distinguished further based on cell morphology and histological staining: LUAD typically appears glandular, whereas LUSC harbours large polygonal cells with squamous differentiation. Different marker proteins also aid diagnosis as TTF-1/NKX2-1 and KRT7 expression are indicative of LUAD, whereas TP63 and KRT5/6 expression are indicative of LUSC [5]. At the genomic level, the mutational and copy number landscapes of LUAD and LUSC are distinct [6–8]. In LUAD, the occurrence of oncogenic driver mutations in *KRAS*, *EGFR*, *HER2*, *MET* and *FGFR1/2*, as well as oncogene fusions involving anaplastic lymphoma kinase (*ALK*), the *ROS1* receptor tyrosine kinase (RTK), neuregulin 1 (*NRG1*), neurotrophic tyrosine kinase receptor type 1 (*NTRK1*) and *RET*, offer possibilities for new targeted therapies. Recent progress has been made using small-molecule inhibitors to target difficult-to-drug mutated forms of KRAS, and clinical trials are on-going [9]. While LUSC is not characterized by the same mutations as LUAD and has fewer targetable oncogenic drivers, tumour suppressor alterations such as *TP53*, *CDKN2A* and *KEAP1* are common in both subtypes [6–8].

Lung cancer survival is highly stage dependent; in England between 2013 and 2017, diagnosis at stage I was associated with a greater than 50% 5-year survival, whereas if the diagnosis was at stage IV, i.e. metastatic disease, the equivalent figure was approximately 3% (Office of National Statistics, UK). In NSCLC, independent of histological subtype, the standard first-line treatment for patients with stage I–III tumours is surgical resection, with adjuvant chemotherapy offering a small benefit for those with locally advanced stage III disease [10]. If surgery is not possible or is declined then chemoradiotherapy is typically offered. Immune checkpoint inhibitors have revolutionized NSCLC treatment and emerging survival data from early phase clinical trials indicate a significant increase in median overall survival for a subset of patients. Anti PD-L1 and PD-1 therapies have been licenced for use in both locally advanced and advanced cases, respectively [11] and although treatment efficacy has been linked to tumour PD-L1 expression [12], patient stratification for immunotherapy agents requires further refinement [13]. Targeted therapy has predominantly focussed on inhibiting the constitutive activation of mutated forms of the epidermal growth factor receptor (EGFR). A majority of patients initially respond to treatment but eventually progress as therapy resistance develops [14]. The emergence of resistance coupled with a high number of unknown resistance mechanisms indicates the potential for rapid tumour evolution [15].

The recent advancements in cancer treatments outlined above would not have been achieved without experimental models to investigate the different aspects of disease initiation and progression. Pre-clinical models represent important tools that allow us to study tumour evolution in the absence of therapy in a manner that is not possible in patients. Along with enabling studies of early disease, these models also allow us to compare the efficacy of novel therapies with established treatments and to study mechanisms of therapy resistance. Such systems have the potential to identify biomarkers of response for patient stratification and to inform future personalized therapies. In this review, we describe the progress that has been made to diversify the tools available for NSCLC research, discuss their relative advantages and disadvantages for particular research questions and reflect on some of the outstanding questions facing the field.

## 2. Pre-clinical NSCLC model systems

**Figure 1.**
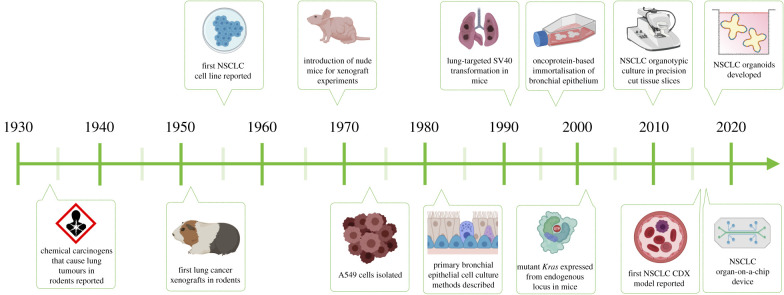
A timeline of advances in pre-clinical models of non-small-cell lung cancer. Created with BioRender.com based on [16–28].

The study of NSCLC has progressed tremendously since the initial investigations identifying chemical carcinogens as a source of lung cancer (figure 1). The technical and scientific advancements in NSCLC research have included the establishment of immortal cell lines, primary cell cultures, xenografts and mouse models, which each have their relative merits and disadvantages (table 1).

**Table 1.** An overview of the relative merits of NSCLC models.

source material	advantages	disadvantages
cell lines	large number available inexpensive and widely available many are well-characterized permissive of genetic manipulation permissive of biochemical studies and drug screens	cell selection based on growth on culture conditions phenotypic changes caused by immortalization divergence/genetic drift may affect reproducibility few modern LUSC lines germline data generally unavailable
primary patient samples normal tissue tumour biopsy circulating tumour cells	close-to-patient: recent clinical data and other matched patient samples permissive to the on-going development of novel models, e.g. those involving organoids and/or co-culture	difficult to maintain AT2 phenotype in culture difficult to establish pre-invasive or LUSC cultures contaminating normal airway cells common in tumour cultures establishment within a clinical timeframe is challenging limited availability
genetically engineered mouse models (GEMms)	reproducible within pure background strains *in situ* microenvironment control over genetic alterations immune competent animals	strong oncogenic drive limits study of early tumour evolution more rapid progression than in human patients multiple allele generation can require many generations and be costly species differences
carcinogen-induced tumour models	mimic pre-invasive disease stages *in situ* microenvironment immune competent animals	more rapid progression than in human patients often develop the extensive multi-tumour disease species differences

### 2.1. Established cell lines

The most frequently reported NSCLC laboratory models are cell lines (table 2), which are inexpensive, scalable and widely available [29,30]. Cells, isolated from patients' tumours have been selected for growth, most often on plastic cultureware, in culture medium containing bovine serum. Lung cancer cells were among the first to be successfully cultivated in the laboratory [32] and, to date, over 200 NSCLC cell lines are available in cell line collections [33,34]. Sequencing efforts from the Wellcome Trust Sanger Institute's cell lines project (https://cancer.sanger.ac.uk/cosmic) and the Broad Institute's Cancer Cell Line Encyclopedia (https://portals.broadinstitute.org/ccle) have identified the mutational status for 110 and 136 NSCLC cell lines, respectively. Community resources such as Cellosaurus (https://web.expasy.org/cellosaurus) also collate publicly available cell line information. Most NSCLC cell lines are derived from adenocarcinomas with fewer LUSC cell lines available due to the lack of effective culturing methods. In general, LUSC cell lines tend to be less well-characterized and there are concerns that some cell lines are potentially misattributed due to their *in vitro* similarity to squamous cancers from other organs. NSCLC cell lines maintain some of the fundamental features of the tumours from which they were derived [35] but the most widely used NSCLC cell lines are now several decades post-establishment, limiting the availability of clinical data and modern genetic characterization of the parental tumour, including germline sequencing. It is important to recognize that, due to on-going mutational processes and genomic instability, the divergence of these long-term cultures from the original tumour occur during continued propagation. Additional complexity and irreproducibility is introduced by the different selection pressures applied as multiple laboratories cultivate cell lines with variable tissue culture practices. Consequently, divergent cell growth behaviour [36] and response to therapies [37] have been reported.

**Table 2.** A list of selected, commonly used NSCLC cell lines along with the driver mutations found in each. Oncogene driver information, TP53 status, sex and ethnicity was derived from COSMIC (https://cancer.sanger.ac.uk/cell_lines) and Cellosaurus (https://web.expasy.org/cellosaurus). LUDLU-1 is described as per a published report [31]. WT = wild type.

name	cancer type	tissue origin	driver mutation	*TP53* status	sex	predominant ethnicity
A549	LUAD/carcinoma	primary	*KRAS* p.Gly12Ser (Hom) *STK11* p.Gln37Ter (Hom)	WT	M	Caucasian
NCI-H322	LUAD	primary	unknown	p.Arg248Leu (Hom)	M	Caucasian
NCI-H358	LUAD	primary	*KRAS* p.Gly12Cys (Het)	Loss (Hom)	M	Caucasian
NCI-H522	LUAD	primary	unknown	p.Pro191fs*56 (Het Sanger/Hom Cellosaurus)	M	Caucasian
NCI-H3255	LUAD	primary	*EGFR* p.Leu858Arg (Hom)	c.560-1G>A (Hom)	F	Caucasian
HCC-4006	LUAD	metastasis: pleural effusion	*EGFR* p.Leu747-Glu749del.	WT	M	Caucasian
PC9	LUAD	metastasis: lymph node	*EGFR* amplified, *EGFR* ex19del	p.Arg248Gln (Hom)	M	unknown
LUDLU-1	LUSC	primary	*BRCA1, BRCA2*	p.Arg248Trp (Hom)	M	Caucasian
NCI-H520	LUSC	primary	*ATM* p.Pro383Ala (Het) *CDKN2A* p.Gly45fs*8 (Hom)	p.Trp146Ter (Hom)	M	Caucasian
NCI-H2170	LUSC	primary	unknown	p.Arg158Gly (Hom)	M	Caucasian
SK-MES-1	LUSC	metastasis: pleural effusion	unknown	p.Glu298Ter (Hom)	M	Caucasian
NCI-H647	adenosquamous	primary	*KRAS* p.Gly13Asp (Hom)	c.782+1G>T (Hom)	M	Caucasian
NCI-H1299	lung large cell carcinoma	metastasis: lymph node	*NRAS* p.Gln61Lys (Het)	Loss (Hom)	M	Caucasian
ChaGo-K1	bronchogenic carcinoma	metastatic site: subcutaneous	*ARID1A* p.His684Asp (Het) *RB1* p.Glu837Lys (Hom)	p.Cys275Phe (Het)	M	Caucasian
NL20	human bronchial epithelial cells	normal bronchus	transformed; SV40, LargeT	WT	F	unknown

### 2.2. Patient-derived tissue

#### 2.2.1. *Ex vivo* explant cultures

Small fragments or slices of resected NSCLC tumours can be maintained in the cell culture medium, allowing short-term investigations. Explant cultures were pioneered as ‘histocultures’ in which tumour pieces were supported on collagen-based sponge gels that enabled short-term tumour cell survival and proliferation read-outs [38]. In a recent study, around 70% of NSCLCs were amenable to explant culture [39] and further optimization might be possible by customizing matrix protein composition and/or using the autologous serum in such cultures [40]. Precision-cut lung slices (PCLSs) can be generated by filling tissue with agarose before creating 100–500 µm sections using a tissue slicer or a vibratome. Sections are then cultured in a maintenance medium and, although survival times vary, some cell types can survive for more than 7 days. PCLSs have gained popularity in other chronic lung disease settings [41], although the difficulties of genetic manipulation within such a short timeframe make transformation studies challenging. The surgical resection of primary tumours lends itself to such studies [16] but, in practice, application of this technology in lung cancer studies remains limited.

#### 2.2.2. Primary cell cultures

Primary cell cultures, in either 2D or 3D culture formats, can be derived from cells retrieved by lobectomies, brush or forcep biopsies, from resected or biopsied tumours, or from circulating tumour cells (CTCs) recovered from blood samples. These are distinguished from cell lines by the relative recency of their establishment and, in some cases, a lack of indefinite proliferative capacity, although the long-term culture of primary cells is now possible in some systems without immortalization. Despite the ease of establishing normal airway epithelial cell cultures, pre-invasive [42] and invasive LUSC cells [43–46] are difficult to culture by existing methods, perhaps due to the presence of widespread CNAs [6,47]. Increasing the culture success rate is hampered by a lack of clarity about what tumour (or cellular) features are selected for by *in vitro* culture conditions. Recently, 3D patient-derived organoids (PDOs) have been established [48,49]. Generating pure NSCLC organoid cultures without the presence of contaminating normal airway organoids is a challenge but can be partially resolved by selection for *TP53*-mutant cells using the small-molecule Nutlin-3a [50].

#### 2.2.3. Patient-derived xenograft (PDX) models

In patient-derived xenograft (PDX) models, patient tumour tissue or CTCs are implanted or injected into an immunocompromised mouse host to achieve continued proliferation of tumour cells. This is most frequently done at a subcutaneous site but can also be orthotopic or injected into the circulation. PDX models are close-to-patient and have a 30–40% success rate [51]. Higher stage tumours engraft more readily in mice and successful engraftment is a negative prognostic indicator in early stage disease [52,53]. While most PDX models are established subcutaneously for convenient tumour burden monitoring, there is evidence that orthotopic [54] or renal capsule [55] transplantation might substantially increase engraftment. Genome editing technologies are also increasingly applicable to xenografts [56]. Nevertheless, the use of a mouse host is a limitation in terms of the stromal, vascular and immune microenvironments experienced by the tumour and, comparable to cell cultures, these models are susceptible to some degree of genomic divergence due to on-going evolution [37].

### 2.3. Model organism research

#### 2.3.1. Genetically engineered mouse models

Most NSCLC model organism work has focussed on the mouse as a result of the powerful genetic tools available for tumour induction and lineage tracing. Genetically engineered mouse models (GEMMs) are typically inbred mouse strains that have been genetically manipulated to express oncogenic alleles or delete tumour suppressor genes to generate autochthonous tumours.

Since smoking-induced lung cancer has a high mutational burden, it is advantageous to use reductionist GEMMs— which allow experimental control over a small number of genetic alterations—to establish which events drive cancer and which are passenger mutations. Simplified genetic models with alterations to a few typically strong oncogenes or tumour suppressor genes are useful to dissect complex pathological mechanisms and test putative therapies in controlled conditions. The conditional mutagenesis systems CRE-LOX and FLP-FRT allow temporal control of genetic events within specific lung cell populations [57]. Still, complex models containing five or more mutated alleles are costly and still lack pre-cancer evolutionary context. While GEMMs have traditionally taken a long time to derive, the emergence of CRISPR–Cas9 genome editing increasingly allows faster derivation [58]. Overall, GEMMs typically provide rapidly developing lung cancer models that generate multiple small tumours in the lungs but, given that they lack the genomic damage caused by tobacco smoking, they often do not capture the mutational diversity of human tumours [59,60]. Incorporating additional mutagenesis, caused either by chemicals or the expression of proteins known to cause mutations, such as cytosine deaminases, might bring mouse models closer to human tumour mutational burden. Importantly, the short (approx. 2 year) lifespan of mice and differences in cell-intrinsic factors, such as in telomere biology [61], mean that GEMM tumours are not subject to the same evolutionary histories as human tumours. Nonetheless, the use of GEMMs has significantly increased our understanding of NSCLC, both with regards to disease progression and potential treatments.

Most NSCLC GEMMs result in adenocarcinoma and there has been a particular focus on those caused by *Kras* mutations. Multiple mutant alleles exist but the most widely used is *Kras*-G12D. Adenocarcinomas can be produced by *Kras*-G12D expression in AT2 cells expressing either *Sftpc* or *Scgb1a1* (*CCSP*; *CC10*) [62]. Although *Scgb1a1* is expressed in bronchiolar club cells in this model, they do not form invasive cancers [62] unless *Trp53* mutations are introduced [63,64]. Lineage tracing in a *Kras*-G12 V model further showed that many alveolar cells expressing the mutant allele do not divide [65], suggesting heterogeneity among AT2 cells. Indeed, during homeostasis, only a subpopulation of AT2 cells are Wnt active stem cells within a fibroblastic niche [66,67] and Wnt signalling has been implicated in the progression [68] of *Kras*/*Trp53* adenocarcinoma models [69]. Multiple other *Kras* combinations have been generated, including those with activating PIK3CA mutations [70] or *MYC* overexpression [71]. Similarly, mouse models of targetable mutations have been developed by expressing human EGFR [72], EML4-ALK fusion kinase [73] or ROS1 fusion kinases [74,75].

LUSC GEMM development has been hindered by the rarity of activating oncogenes, the lack of lung basal cell-specific Cre-drivers and the absence of basal cells from airway epithelium distal to the main bronchi in mouse (compared to humans, where they are present throughout many airway generations). Combining *Sox2* overexpression with tumour suppressive *Pten* and *Cdkn2a* mutations leads to LUSC-like tumours regardless of whether the Cre-driver gene is expressed by basal, club or AT2 cells [76]. *Sox2* overexpression in mouse club cells leads to the proximalization of the bronchiolar epithelium and adenocarcinomas expressing the squamous marker TP63 eventually form [77]. Hybrid approaches using mouse genetics and *in vitro* organoids have also allowed the development of tumorigenic mouse organoids that overexpress *Sox2* and harbour deletions in the key LUSC tumour suppressor genes *Trp53*, *Cdkn2a* and *Pten* [78]. *Lkb1* loss, a rare event in human LUSC, together with *Sox2* overexpression lead to mouse LUSC [79]. In LUAD, the addition of *Lkb1* mutations to *Kras*-driven GEMMs has confirmed that they modify histology, as dual mutants give rise to LUAD, LUSC and mixed adenosquamous lesions [80]. In established *Kras*-driven tumours, *Lkb1* loss promotes the transition to squamous histology [81] with redox balance [82] and epigenetic mechanisms involving polycomb repressive complex 2 (PRC2) [81] both implicated mechanistically in LUSC formation.

#### 2.3.2. Carcinogen-induced NSCLC

Exposure of rodents to particulate matter, whole tobacco smoke or e-cigarette vapour produces physiologically relevant changes in the lung epithelium [83,84] and accelerates tumour development [85–87]. Cigarette smoke condensate or pure chemical carcinogens induce lung cancers that arise through relevant pre-invasive disease processes. The most commonly used, urethane, generates predominantly *Kras*-driven LUAD which transitions through adenoma precursor lesions [88]. Multiple carcinogen-driven LUSC models are also available: benzo[a]pyrene causes squamous bronchial lesions in hamsters [89]; repeated intra-tracheal injection of 3-methylcholanthrene (MCA) in mice causes metaplastic lesions throughout the bronchial tree which progress to invasive and metastatic LUSC [90]; and N-nitroso-tris-chloroethylurea (NTCU) produces a LUSC-like lung cancer when applied to the back skin of mice [91–93]. Susceptibility to chemical carcinogens varies by mouse strain and correlates with the prevalence of spontaneous tumour formation.

## 3. Cancer origins in ‘normal’ tissue

The use of clonal cell culture to expand single progenitor cells has allowed whole-genome sequencing of histologically normal airway epithelium [42], revealing the presence of somatic mutations, including known cancer driver mutations. As expected, the mutational burden increased with age and was significantly higher in adults with a tobacco smoking history [42]. The detected cancer driver mutations were those typical of LUSC, with *TP53*, *NOTCH1* and *FAT1* mutations being the most frequent. Individual cells rarely contained multiple driver mutations but, unlike in LUSC tumours, copy number alterations were uncommon [42]. It remains unclear whether primary ‘normal’ airway epithelial cell culture imposes selective pressures similar to those observed in established cell lines [36,37]. Despite these concerns, primary airway cultures might facilitate studies on differences in cancer susceptibility among the four recently identified subpopulations of cultured normal airway basal epithelial cells [94].

Airway basal cells have also been immortalized, for example by using overexpression of CDK4 and TERT [95], and region-specific differences in airway biology are retained in cell lines from different locations in the proximal–distal axis of the bronchial tree [96]. To more closely resemble fully differentiated airway epithelium, it is possible to direct basal cell differentiation towards multiciliated and mucosecretory cell fate in either air-liquid interface cultures [97] or as 3D ‘tracheospheres’ [98]. More recent advances in organoid culture enable expanding cultures of airway epithelial cells containing all three major cell lineages as 3D organoids [48].

Unfortunately, *in vitro* studies of primary alveolar epithelial cells—and therefore efforts to map mutations in the normal alveolar epithelium—have been hindered by the short time that it is possible to maintain proliferating AT2 progenitor cells in culture before they differentiate to non-proliferative AT1-like cells [99]. Immortalized alveolar epithelial cells have been developed [100] but the narrow timeframe available for transduction with immortalization factors means that, similar to primary alveolar cultures, these cells more closely resemble AT1 cells. Use of Rho-associated protein kinase (ROCK) inhibitors during AT2 cell culture derivation might enable AT2 cell-like phenotype preservation for studies of step-wise carcinogenesis [99]. Similar to 2D cultures, most reported alveolar organoid systems have not allowed serial passaging [67,101], limiting their utility for cancer modelling. Recently, long-term culture methods for human organoids containing AT2 cells that are capable of AT1-like differentiation have been described [102,103], further expanding the repertoire of models available.

Robust and scalable primary airway and alveolar culture methodologies have the potential to improve our understanding of the effect of specific somatic mutations on cellular dynamics in human epithelia and are compatible with CRISPR–Cas9-mediated gene editing approaches that might allow temporal reconstruction of NSCLC molecular events, as has been possible in studies of colorectal carcinogenesis [104].

## 4. Modelling early tumorigenesis

Early detection of NSCLC is a priority for improving clinical outcomes, particularly in cases where intervention could occur before invasive disease occurs. Achieving this will require a better understanding of pre-invasive cancer biology but the required laboratory studies are currently challenging. GEMMs typically introduce strong oncogenic and/or tumour suppressor alterations in many cells simultaneously, thus limiting the extent to which their progression mimics early tumour evolution. Nevertheless, mouse models allow a high degree of genetic control and provide a much greater range of tumour microenvironmental cues than culture systems. Primary or immortalized cell cultures from carcinoma-in-situ or adenomatous lesions have not yet been derived but progression to malignancy can be investigated through the introduction of cancer mutations into immortalized ‘normal’ basal cell lines [105]. This approach has identified a subpopulation of basal epithelial cells with enhanced motility [106] and has been used as an organotypic model of dysplasia by introducing *TP53* inactivation and *SOX2* overexpression [107]. It is highly likely that both GEMMs and cell cultures will be invaluable tools for establishing the physiological order of events that drive early oncogenesis and the dependencies of pre-malignant cells above and beyond those of normal tissues.

Chemical carcinogen models are particularly relevant in early tumorigenesis research because the tumours undergo a histological transition similar to that seen in human patients. In the urethane-induced LUAD model, the tumours are also morphologically similar to spontaneous tumours in aged mice, with a robust immune infiltrate organized in tertiary lymphoid structures. This observation, together with the fact that the growth pattern of LUAD tumours varies between chemicals, suggests that carcinogens accelerate physiologically relevant processes [108,109]. Consistent with patient data [110], *Kras* mutations are identified as an early event, *Cdkn2a* can be epigenetically downregulated in early foci and later deleted in both adenomas and adenocarcinomas, while *Trp53* mutations are found in adenocarcinomas but not hyperplasias, supporting a role for *Trp53* in invasion [60]. However, it is noteworthy that *Kras* G12C mutations are not found in either urethane- (Q61R/Q61 L) or nitrosomethylurea-induced (NMU; G12D) [60] mouse tumours as these carcinogens do not induce the requisite base substitution.

Squamous chemical carcinogen models also mimic patient pre-cancerous lesions, albeit on an accelerated time frame. In NTCU-treated mice, tracheal dysplasia precedes proximalization of the bronchial epithelium and progression to invasive LUSC [111]. In spite of the differences in cellular composition between mouse and human airways [112], the model recapitulates key aspects of LUSC natural history, with KRT5-expressing lesions and apparent PI3 K signalling [113]. RNA sequencing analysis suggests similarities in the immune response of LUSC patients and NTCU-treated mice [114], although full genomic characterization to compare mouse to human lesions is not yet available and differences emerge between mouse strains and sexes [115]. Despite some systemic and local side effects, NTCU-treated mice rarely develop other squamous cell carcinomas, such as of the skin or oesophagus, and are suitable for chemoprevention studies. Therefore, deeper characterization and comparison of carcinogen-induced NSCLC models using modern genomic, epigenomic and transcriptomic tools are warranted.

## 5. Drug response

Cell lines are extensively used for assaying drug efficacy as clonal genetic alterations are maintained and they are amenable to high-throughput assays. There are concerns about genetic and functional differences between different sublines of established cancer cell lines, including A549 cells [37], that might result in divergent responses in compound screening experiments [116]. The use of cell lines to predict the efficacy of treatment in relation to particular tumour features, such as histology or particular mutations, will probably require investigating a large number of cell lines in order to generate robust data. Efforts such as the Cancer Cell Line Encyclopaedia [117] that have deeply characterized NSCLC cell lines enable the integration of genetic characteristics with functional drug sensitivity assays (e.g. those that can be interrogated via DepMap; https://depmap.org/portal). Patient-derived models such as primary cell cultures, PDOs and PDX models are expected to display greater fidelity to the behaviour of the patient tumour as a result of the recency of establishment. Indeed tumour explants [38] and PDX models [118] can be predictive of the efficacy of chemotherapy, while PCLSs have also been used to test novel therapeutics [119]. Comparisons of pre-clinical model efficacy with phase II clinical trial results support the predictive value of cell lines and human xenografts but not mouse allografts in NSCLC [120]. Overall, these studies show that selecting a range of cell lines (or patient-derived models) to address specific questions (e.g. within mutation status or histology) can improve predictivity and argue the need for comparing multiple well-characterized cell lines with other disease models.

Pre-clinical models are not only important tools for predicting the efficacy of targeted therapies, but also for determining mechanisms of resistance, for example to chemotherapies or targeted therapies. That cell lines can develop resistance to therapies through the expansion of pre-existing subclones argues that they preserve some tumour heterogeneity [121]. A concern is that selection pressure upon cell line establishment or during expansion might not fully reflect the primary cancer, thus preventing the discovery of possible resistance mechanisms. These concerns are also relevant in patient-derived models, reiterating the importance of using a wide range of models and maximizing comparisons with patient datasets where these are available.

In addition to predicting cohort-level responsiveness to therapy, it has also been proposed that patient-derived models might predict drug efficacy for individuals. However, for precision oncology to become a reality, the scalability and time frame must fit the clinical need. Currently, these models take several weeks or months to establish, but in one study reductions in the time required to get clinically actionable information from PDX models was achieved by implantation of tumour pieces in the subrenal capsule followed by testing of alternative chemotherapy regimens with results known within eight weeks [55]. Although not yet applied to NSCLC, patient-derived tumour cell clusters have been developed which allow short-term cell expansions from primary tumours, including immune and fibroblast populations, allowing the investigation of hundreds of therapeutic options per patient in a manner that correlated with the clinical performance [122]. Of course, a caveat of these strategies might be tumour complexity with regards to intra-tumour heterogeneity and sampling bias. This concern might be minimized by targeting truncal mutations and perhaps by using as much as possible of the tumour material remaining after clinical diagnostics to generate more representative models [123].

## 6. Tumour microenvironment

An ideal model of the tumour microenvironment would comprise patient-matched tumour, stromal and immune cell populations with their native architecture preserved. Tumour explant cultures and PCLSs attempt to achieve this by maintaining living, surgically resected tumour tissue in the laboratory. Although existing studies largely focus on tumour cells in these systems, such cultures also offer an opportunity to study tumour–stroma interactions and the effects of immunomodulatory drugs. T cell populations can be imaged within PCLSs [124] suggesting the opportunity to study cellular localization, for example, the exclusion of T cells from tumours [125], in a manner that is not possible in other human *in vitro* systems. Nevertheless, the limited timeframe of such experiments and the differential sensitivity of different cell types to culture mean that mouse models and reductionist human models recapitulating specific cellular interactions remain the mainstay of tumour microenvironment research in NSCLC.

### 6.1. Tumour–stromal interactions

Increasingly, we recognize the importance of cancer-associated fibroblasts (CAFs) as regulators of lung cancer growth, immunogenicity and metastasis. CAFs are fibroblasts that have been activated by a variety of signals in the tumour microenvironment and single-cell RNA sequencing studies suggest the heterogenous nature of lung CAFs [126].

Genetic and syngeneic mouse models feature authentic stromal–epithelial interaction after tumour induction allowing the study of the interplay between the tumour and stroma. In xenotransplantation experiments, host–tumour interactions can be limited by cell signalling incompatibilities between species. A prominent example is stromal mouse hepatocyte growth factor (HGF), which cannot fully activate the human MET receptor in epithelial cells [127], potentially limiting the growth of HGF-dependent tumours. However, human fibroblasts are readily expanded in cell culture on plastic, allowing comparisons between healthy control, matched adjacent lung and tumour-associated fibroblasts. These can be introduced into cell lines, primary cell culture or xenograft assays to determine phenotypic changes and their effects on epithelial cells. Experiments to date support the notion that (at least a subset of) CAFs act as a supportive niche that maintains NSCLC cells in a de-differentiated state [128–130]. Fully describing CAF subsets, the epigenetic stability of the CAF state and the extent to which targeting CAFs can be therapeutically beneficial are active areas of research [131].

### 6.2. Mechanical force

Advancements in microfluidic technologies have enabled the development of NSCLC cancer-on-a-chip devices. Originally these devices were designed as a ‘lung-on-a-chip’, combining airway or alveolar epithelial cells with endothelial cells in distinct channels [132]. When the EGFR-mutant LUAD cell line H1975 was seeded in this system among normal epithelial cells, it grew faster among alveolar than airway epithelial cells [17], consistent with its alveolar origin. Application of mechanical force to mimic breathing led to reduced proliferation of the cancer cell line, which also became more resistant to the EGFR inhibitor rociletinib [17], demonstrating the potential for changes in cell behaviour upon incorporating physiological parameters into cell culture experiments.

### 6.3. Tumour-immune interactions

In lung cancer immunology research, intact immune surveillance is available in GEMMs as well as carcinogen-induced and syngeneic mouse models. Since immune evasion occurs early in LUSC pathogenesis [133,134], carcinogen-based models might offer an opportunity for immunotherapy development. Three main syngeneic models have been described: Madison 109 (MAD109) was derived from a spontaneous BALB/c mouse lung tumour in 1964 [135], the Lewis lung carcinoma (LLC) cell line was derived from a C57BL mouse in 1951 [136] and KLN-205 was derived from a DBA/2 mouse treated with MCA in the 1970s [90,137]. These models produce very rapidly growing tumours in the lung either following direct implantation or as a result of metastases from subcutaneous xenografts. As discussed above, GEMMs are frequently poorly immunogenic, presumably due to their low mutational burden. Nevertheless, both syngeneic models and GEMMs can be modified to present model antigens such as ovalbumin in order to study antigen presentation and recognition by transgenic T cells engineered with reactivity against ovalbumin-derived peptides [138,139]. More recently, the inversion-induced joined neoantigen (NINJA) system has been developed, which allows spatially and temporally controlled expression of defined neoantigens in mouse lung cancer cell lines, lung tissue and in GEMMs [140]. Crucially the system avoids central tolerance and as such will allow the investigation of endogenous T cells in anti-tumour immunity [140].

Nevertheless, there are substantial differences between the composition, protein production and function of the human and mouse immune systems. Of particular relevance given that neutrophils are the most abundant immune cell population in the NSCLC tumour microenvironment [141], human blood contains more neutrophils than lymphocytes, whereas the mouse lymphocytes substantially outnumber neutrophils [142]. As such, a range of model systems now exist to explore human immune cells in the context of NSCLC.

Firstly, human immune cells can be introduced into mouse models. In adoptive cell therapy experiments, injection of tumour-bearing mice with engineered CAR-T cells has expanded studies beyond *in vitro* reactivity assays [143] and could similarly be applied to tumour-infiltrating lymphocytes (TILs) [144], which have entered early phase clinical trials for NSCLC. To investigate checkpoint inhibitors, TILs within initial biopsies can be studied by co-administering checkpoint inhibitors at the time of tumour implantation and then sacrificing the mice at early time points [145]. Although the long-term effects of therapy on tumour burden cannot be established in this model, it is nevertheless possible to study the phenotypes of both the tumour and immune cell populations.

In xenotransplantation experiments, the required use of severely immunocompromised mice with deficiencies in both innate and adaptive immune responses has drawbacks for studying pathogenesis and testing potential therapies, particularly emerging immunomodulators. More complete reconstitution of the human blood and immune cell lineages has been achieved in ‘humanized’ mice where haematopoietic stem cells (HSCs) are engrafted in NSG mice following irradiation-induced clearance of host progenitor cells [146]. Humanized mice can live for more than 30 weeks post-transplantation [147], which has allowed the subsequent application of a human tumour model. The response to anti-PD-1 therapy was dependent upon the presence of human T cells and variable depending on the donor cells used for reconstitution [148]. Unfortunately, it is rarely possible to isolate HSCs and tumour cells from the same individual and, even in donor-matched experiments, the T cells that develop in humanized mice are educated in the mouse thymus and thus selected on mouse MHC, resulting in an allogeneic response which limits studies of tumour-specific antigen responses.

In studies of newly derived cell lines or PDOs, it is possible to establish simultaneous patient-matched cultures of fibroblasts or immune cells to increase model complexity and better represent the tumour microenvironment. For example, T cell-tumour interactions have been investigated in entirely *in vitro* systems with expansion of tumour-reactive T cells from PBMCs and co-culture with autologous NSCLC organoids [18].

## 7. Metastasis and relapse

**Figure 2.**
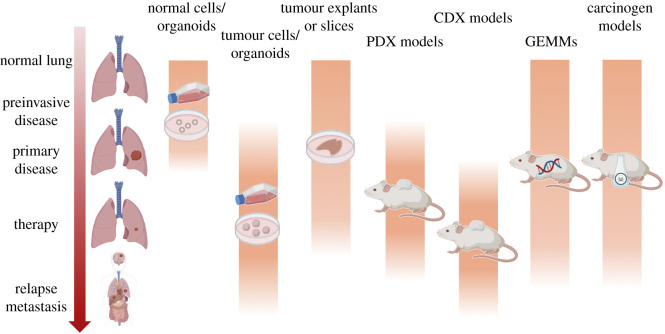
Pre-clinical models across the disease course of non-small-cell lung cancer. Created with BioRender.com.

NSCLCs commonly metastasize to the brain, bone, liver and adrenal glands [149]. Compared to primary disease, tissue acquisition in metastatic, progression and relapse settings are more challenging; clinical diagnosis typically relies on imaging and biopsies, but metastases are rarely surgically resected. Some NSCLC cell lines have been derived from metastases but most lung PDX models are primary tumour-derived even though metastases have a high engraftment rate (figure 2) [150].

CTCs, which are more abundant in patients with late-stage cancers [151], might help to overcome the primary disease bias of PDX collections. Methods to derive CTC-derived explant (CDX) models have been developed in SCLC [152] where researchers faced similar sampling difficulties. CTCs are more frequent in SCLC than in NSCLC patients, and while NSCLC CDX models have been reported that mimic donor patient treatment response [19,153], the success rate is considerably lower than for SCLC using current methods. A marker-independent methodology to isolate CTCs for engraftment is especially important in NSCLC as the partial epithelial-to-mesenchymal transition may result in loss of the epithelial CTC capture and detection markers commonly used in some CTC platforms. Short-term CTC cultures are emerging as alternatives to study the biology of metastatic disease [154]. Another method has been to culture metastatic NSCLC cells from malignant pleural effusions before injection as xenografts [155]. Lung cancer metastasis can also be comprehensively characterized in autopsy settings but there are obvious challenges in maintaining cell viability for the establishment of pre-clinical models, with key parameters including the intervals to both refrigeration and to sampling. Nevertheless, early reports suggest that model derivation is possible through rapid autopsies (ideally performed within 24 h post-mortem) in both primary NSCLC and in the metastasis of cancers from other organ sites to the lungs [156].

The injection of cancer cell lines into immunocompromised mice is an extensively reported *in vivo* cancer metastasis model [157]. These experiments are particularly relevant in lung cancer because cells injected into the tail vein circulate to the lungs, providing a model of metastasis when cells become lodged in small alveolar capillaries. However, such experiments are limited by the drawbacks of prior culture and additional issues caused by a second bottleneck imposed by an introduction to the mouse circulation and the fast-growing and homogeneous nature of the resulting tumours. Brain metastases can be initiated through intracranial or intracardiac delivery of cells in mice and more advanced models—in which human brain metastasis-initiating cells are injected via the intrathoracic route, reform lung tumours and subsequently re-seed brain metastases [158]— have focussed on the earliest steps of brain metastatic colonization with the rationale that future therapies might prevent the seeding of metastases before they form.

Neither *Kras* G12D- or *Egfr* L858R-driven LUAD GEMMs generate robust spontaneous metastasis [59], although the introduction of *Trp53* loss into the *Kras* G12D model is able to generate metastatic lesions [69,159,160]. It was recently shown that mice with additional E-Cadherin loss suffer an increased incidence of metastasis to the chest wall, lymph nodes, liver and kidney [161]. The development of new barcoding technologies, such as high-throughput barcode sequencing (Tuba-seq) [162,163], coupled with CRISPR–Cas9-mediated gene editing, will facilitate studies to determine the effects of other additional mutations on tumour evolution and to identify pro-metastatic factors [164].

It is noteworthy that the *Kras* G12D-driven GEMM also releases tumour-derived cell-free DNA (cfDNA) into circulation [165] which might provide a tractable model to study mechanistic questions about cfDNA release as liquid biopsies move towards clinical application in the early detection and relapse settings. Recently, zebrafish have emerged as a powerful alternative to mouse metastasis and drug sensitivity models as they require less patient material, are more rapidly established and their near transparency as embryos and larvae facilitates imaging studies [166]. However, concerns regarding species differences increase with evolutionary distance.

## 8. Future directions

Despite extensive progress in established and new NSCLC pre-clinical model systems, there remain a number of questions for the field to reflect upon (table 3). Pre-clinical models are unevenly distributed across the disease course, with an overwhelming majority representative of primary NSCLC and relatively few available in the pre-invasive, metastatic, progression or relapse settings. Efforts to prioritize early detection in lung cancer are likely to lead to increased diagnosis of pre-invasive and early stage disease, making new and improved experimental systems to model and predict the trajectory of pre-cancerous lesions invaluable. An expanding knowledge of the mutations and copy number events that are tolerated and positively selected within physiologically functional epithelium will also help to guide more sophisticated models of early NSCLC.

**Table 3.** Outstanding questions in the application of pre-clinical model systems to non-small-cell lung cancer.

questions regarding the use of pre-clinical models	discussion points
why is there a low success rate in translatability of pre-clinical data to clinical applications?	do we do enough orthogonal validation of pre-clinical model findings in patient samples? statistically significant differences observed *in vitro*/*in vivo* might not translate into biological relevant differences in patients pharmacokinetic differences between species
how does the fact that genetically complex GEMms traditionally lose or gain all of their modifications at the same time point impact their tumour evolution?	in human disease, subclonal modifications occur later during disease progression better models with temporal modifications are needed in order to model acquired metastatic potential we typically induce tumours in young mice, does age affect temporal evolution?
how much does pre-clinical model choice skew research outcome?	do we fully understand the extent to which drug responses *in vitro* are shaped by cell culture artefacts (e.g. genetic drift, culture conditions)? how would existing NSCLC therapies perform across an unbiased array of pre-clinical models? how should we weight evidence from different models where data are contradictory? are patient sex and ethnic background sufficiently explored as variables?
can we model copy number-driven tumours in non-human species?	genome organization across species is not conserved so tumour copy number evolution will be dissimilar
are the molecular mechanisms leading to drug resistance shared between pre-clinical models and patients?	species differences and non-physiological conditions might lead to resistance mechanisms not observed in the clinic
how should we use knowledge from big datasets to generate hypotheses that can be investigated in pre-clinical models?	do existing training programs sufficiently emphasize computational skills?
are the clinical parameters investigated during drug screening in agreement between species?	tumour burden monitoring and definitions of progression vary between models and patients species differences might exist in the side effects profiles of new therapies

In addition to deriving new models, there are opportunities to improve the efficiency and reproducibility of existing approaches. Creating and distributing cell lines, GEMMs and xenograft models that generate reproducible results is a priority. In doing so, effort should be made to ensure that patient-derived models are from well-characterized patients with in-depth molecular and genetic characterization and high-quality control standards. For example, human epithelial cell markers should be monitored in PDX models as xenografts can form human lymphomas or mouse sarcomas [167], while primary cell cultures should be monitored for contamination with non-tumour stromal or epithelial cells.

Care should also be taken to address biases in our development of patient-derived models relating to patient sex, ethnicity and cancer genomics. Most pre-clinical models in Western countries are derived from male Caucasian or Hispanic patients, with few from patients of other ethnicities, and they typically represent smoking-associated NSCLC. For example, patients of East Asian descent constitute only 2% of NSCLC cell lines in US-based collections [33], meaning that never-smoker, EGFR-driven NSCLC is underrepresented. Effective international efforts to share pre-clinical models are required to help to address these issues and allow the collection of sufficient numbers of well-described models for more diverse patient groups and those with rare mutations.

Finally, the design of NSCLC animal experiments might also benefit from mimicking treatment regimens and study designs that are used in clinical practice. Commonly used endpoints in clinical studies—such as overall survival, progression-free survival, time to progression and overall response rate—map poorly to studies in mice. This is particularly true when using subcutaneous tumour/cell lines xenografts whose growth have limited systemic impact. GEMMs might be amenable to more comparable dosing, dose regimens and sequential therapeutics [168]. Mouse studies might also more closely mimic RECIST guidelines in which a partial response is defined as a 30% loss and progressive disease as a 20% increase in the sum of lesion diameters against baseline [169]. The study of progression, metastasis and relapse is also complicated by the divergent approaches taken in mouse and human studies. Surgical resection of primary tumours in rodent models is challenging, particularly due to multifocal tumour growth. Indeed, even when this is less problematic, such as in breast cancer models [170], the long and variable latency of metastatic lesion appearance creates challenges for timing therapeutic interventions in most current mouse models.

## 9. Conclusion

In the past several decades, our understanding of NSCLC has moved from histopathological depictions, through an increased comprehension of molecular and genetic causes towards understanding the effects of the tumour microenvironment and the molecular dynamics of tumour evolution. Pre-clinical lung cancer research continues to become more multidisciplinary with contributions from the fields of developmental biology, stem cell biology and immunology helping to decipher interactions between tumour cells and their environment. In addition, it is vital that new clinical knowledge is fed back to improve pre-clinical models. While no models are (or are ever likely to be) able to fully recapitulate the phenotypes and responses of patient tumours, the application of multiple approaches with an awareness of their limitations is driving progress in the field of NSCLC.

## Data accessibility

This article has no additional data.
